# RNA in situ hybridisation as a molecular diagnostic technique targeting IBA‐1 and CD204 in canine histiocytic sarcoma

**DOI:** 10.1002/vms3.795

**Published:** 2022-03-26

**Authors:** Julia L Engelien, Amandine T Lejeune, Michael J Dark, Rowan J Milner, Keijiro Shiomitsu

**Affiliations:** ^1^ Department of Small Animal Clinical Sciences and Comparative Diagnostic and Population Medicine College of Veterinary Medicine University of Florida Gainesville Florida; ^2^ Department of Surgical and Radiological Sciences, School of Veterinary Medicine, University of California, One Shields Avenue, Davis, CA 95616.

**Keywords:** diagnosis, dog, histiocytic disorder, ionised calcium‐binding adapter molecule 1, macrophage scavenger receptor, RNAscope

## Abstract

**Background:**

Canine histiocytic sarcoma (HS) is an aggressive cancer with morphologically variable features; therefore, obtaining a definitive diagnosis can be challenging. Two proteins, IBA‐1, ionised calcium‐binding adapter molecule 1, and CD204, a macrophage scavenger receptor, have been shown to be specific immunohistochemical markers helpful in distinguishing HS from other tumour types with similar morphological features.

**Objectives:**

This study was performed to demonstrate the use of RNA in situ hybridisation (ISH) technology allowing single‐molecule RNA visualisation in formalin‐fixed paraffin‐embedded (FFPE) tissues as a molecular tool for the diagnosis of canine HS.

**Methods:**

Reverse transcription polymerase chain reaction (RT‐PCR) and western blot analysis for IBA‐1 and CD204 were performed to correlate gene expression and protein expression of these two markers in the histiocytic sarcoma DH82 cell line. RNA‐ISH for IBA‐1 and CD204 was performed on the DH82 cell line to validate the RNA‐ISH probes. RNA‐ISH and immunohistochemistry (IHC) were performed in clinical HS FFPE samples to demonstrate mRNA and protein expression of IBA‐1 and CD204. FFPE archived samples of canine round cell tumours, melanoma and anaplastic sarcoma were used as negative controls.

**Results:**

RNA‐ISH and IHC showed moderate to strong expression for IBA‐1 and CD204 in the neoplastic cells in both the canine DH82 cell line and the archived canine HS samples. RNA‐ISH and IHC showed scattered positive staining in the control tumours samples, consistent with macrophagic infiltration.

**Conclusion:**

RNA‐ISH for CD204 and IBA‐1 appeared to have a high specificity and sensitivity in our samples and may be an additional valuable diagnostic technique in identifying HS.

## Graphical abstract

Canine histiocytic sarcoma (HS) is an aggressive cancer with morphologically variable features; therefore, obtaining a definitive diagnosis can be challenging. Two proteins, IBA‐1 and CD204, have been shown to be specific immunohistochemical markers helpful in distinguishing HS from other tumour types with similar morphological features. Use of novel RNA in situ hybridisation (ISH) technology for identification of CD204 and IBA‐1 appeared to have a high specificity and sensitivity in our samples and may be a valuable diagnostic technique in identifying HS.

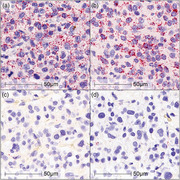

## 1 INTRODUCTION

Canine histiocytic sarcoma (HS) is a highly aggressive neoplasm. It can be characterised into three forms: localised, disseminated and haemophagocytic. Both localised and disseminated HS are derived from an interstitial dendritic cell origin and are thought to be separate stages of the same disease (Affolter & Moore, 2002; Fulmer & Mauldin, 2007; Moore et al., 2006). Haemophagocytic HS is of a macrophagic origin. All three types of HS are derived from the same haematopoietic stem cell precursors (Affolter & Moore, 2002).

HS is difficult to diagnose as its morphological features are highly variable (Affolter & Moore, 2002; Erich et al., 2018; Wakahashi et al., 2010). The morphologic features of HS can often mimic other tumours of mesenchymal or round cell origin such as sarcomas, plasma cell tumour, mast cell tumour, lymphoma, transmissible venereal tumour or amelanotic melanoma. In Erich et al, 47.5% of cases diagnosed with histiocytic sarcoma or its differential diagnoses using histology were reclassified using morphological criteria paired with an immunohistochemistry (IHC) protocol (Erich et al., 2018).

In the past decade, CD204 has been described as a useful marker for identification of cells of macrophagic origin and as a more specific immunohistochemical marker for canine HS (Kato et al., 2014; Kato et al., 2013; Ide et al., 2011; Thongtharb et al., 2016). CD204, or class A macrophage scavenger receptor, is a receptor typically expressed in macrophages. In Kato et al. (2013), CD204 was found to be highly expressed in all HS samples, including those of both macrophagic and dendritic phenotypes. CD204 was not expressed by other round cell tumours or sarcoma samples in this study, except in one grade III mast cell tumour, apart from infiltrating macrophages. In Ide et al, most pleomorphic neoplastic cells in subdural HS expressed CD204 (Ide et al., 2011). Similarly, in Thongtharb et al. (2016), all tumour samples (primary intracranial HS) were found to have a moderate to strong expression of CD204.

Additionally, IBA‐1, or ionised calcium‐binding adapter molecule 1, a protein mediating calcium signals in monocytic and histiocytic cells, has been reported to be a specific diagnostic marker for canine HS (Ide et al., 2011). It is expressed in all cells of macrophage or monocyte origin, including all three forms of HS (Pierezan et al., 2014). In one study, selected cases of histiocytic disease, including canine histiocytomas, reactive histiocytosis and histiocytic sarcomas, were found to have a moderate to strong cytoplasmic immunoreactivity for IBA‐1, whereas samples of melanomas, lymphomas, mast cell tumours and plasma cell tumours were negative, apart from scattered positive non‐neoplastic stromal and peritumoural histiocytes as well as epidermal Langerhans cells (Pierezan et al., 2014). Similarly, all HS tumour samples assessed by Thongtharb et al. (2016) and by Ide et al. (2011) showed a moderate to strong immunoreactivity for IBA‐1.

Therefore, both IBA‐1 and CD204 are considered to be useful, specific markers in the diagnosis of HS by distinguishing it from similar conditions (Kato et al., 2013; Kato et al., 2014; Pierezan et al., 2014).

RNAscope® in situ Hybridisation (ISH) is a technique to evaluate mRNA expression. It allows for visualisation of a single molecule of RNA in both formalin fixed tissues and cytological samples (Bingham et al., 2017; Chan et al., 2018). This technology allows for analysis of mRNA expression without destroying tissue architecture.

The primary goal of this study was to evaluate the expression of IBA‐1 and CD204 in the canine HS cell line DH82 and in archived formalin fixed tissue from patients diagnosed with HS using RNA‐ISH. We assessed the expression and visualisation of RNA using the RNA‐ISH technique to validate a useful, less complicated and definitive diagnostic tool for canine HS. We also assessed IBA‐1 and CD204 protein levels in HS by immunohistochemistry and compared the results with those obtained using RNA‐ISH.

## 2 MATERIALS AND METHODS

### 2.1 Cell culture

The HS cell line DH82 (American Type Culture Collection: ATCC, No. CRL‐10389, Manassas, VA) was utilised to validate mRNA and protein expression of IBA‐1 and CD204. This cell line has been previously described and extensively characterised. The DH82 cell line was established in 1988 from the neoplastic progenitor cells of canine malignant histiocytosis (Asada et al., 2015; Barnes et al., 2000; Heinrich et al., 2015; Wellman et al., 1988)^.^ The DH82 cells were cultured in a T‐75 flask with minimum essential media (MEM: Corning, Manassas, VA) with 10% foetal bovine serum (FBS, Gibco, Waltham, MA) and 1% antibiotic‐antimycotic (Gibico) at 37°C in 5% CO_2_ until 90–100% confluency.

### 2.2 Cell pellets

Cell pellets, derived from the DH82 cell line, were utilised for both immunohistochemistry (IHC) and RNA‐ISH staining. Once DH82 reached 90–100% confluence, cells were trypsinised, transferred into 50 ml conical tubes and then centrifuged at 400 *g* for 10 min. Cell pellets were fixed in 10% neutral buffered formalin, and paraffin embedded pellets were prepared using histogel‐based cell block preparation (HISTOGEL, ThermoFisher Scientific, Waltham, MA) following manufacturer's instructions.

### 2.3 Formalin‐fixed paraffin‐embedded (FFPE) tissue

Archived paraffin‐embedded blocks of 8 canine HS and 10 canine tumour types commonly included in the differential diagnosis (DDx) of HS (oral malignant melanoma *n* = 2, mast cell tumour *n* = 2, lymphoma *n* = 2, plasma cell tumour *n* = 2 and anaplastic sarcoma *n* = 2) were obtained. Histologic sections of tumour were reviewed, and a definitive diagnosis was made by board‐certified veterinary pathologists at one public academic institution. A second review was performed by one single board‐certified veterinary pathologist (M,D) to confirm the diagnosis. The tissues had been fixed in 10% buffered neutral formalin and paraffin embedment following standard protocols. FFPE tissues were sectioned at 5 μm, mounted on positively charged Superfrost Plus slides (Fisher Scientific, Pittsburgh, PA) and deparaffinised.

### 2.4 RNA extraction

RNA extraction from the DH82 cell line was performed using a commercial kit (RNeasy Mini Kit, Qiagen, Valencia, CA) following the manufacturer's instructions. Cells were counted using a haemocytometer, and 3 × 10^6^ cells were used for RNA extraction. Extracted RNA was quantified by a spectrophotometer (Nanodrop 8000, Thermo Fisher Scientific). The ratio of the optical densities at 260 and 280 was >1.9. cDNA synthesis for reverse transcription‐polymerase chain reaction (RT‐PCR) was completed using QuantiTect Reverse Transcription kit (Qiagen). The RNA samples were treated with H_2_O, 1 μg RNA and 1 μl of gDNA wipeout buffer and incubated in a thermocycler (SimpliAmP Thermocycler, ThermoFisher Scientific) at 42°C for 2 min. The samples were treated with 1 μl Quantiscript Reverse Transcriptase, 4 μl Quantscript RT Buffer 5×, 1 μl RT Primer Mix and 14 μl of template RNA from gDNA elimination reaction. Samples were incubated for 30 min at 42°C and 3 min at 95°C in the thermal cycler. cDNA samples were stored at −80°C.

### 2.5 Reverse transcriptase‐polymerase chain reaction (RT‐PCR)

RT‐PCR was performed on the DH82 cell line using primers for GAPDH (reference gene), IBA‐1 and CD204. The primers were generated from information obtained from a genetic sequence database (Genbank). Primer 3 (version 4, http://bioinfo.ut.ee/primer3‐0.4.0/) was used to design primers for canine specific target genes (GAPDH: Accession: NM_001003142.2, IBA‐1: accession: XM_532072.6 and CD204: XM_843168.4). BioEdit (Ver. 7.2) was used to align each sequence (1). The sequences of primers and predicted size applications are listed in Table 1. RT‐PCR was performed by combining 19 μl of RNAse free water, 25 μl of master mix (HotStar Taq, Qiagen, Germantown, MD), 2 μl of cDNA generated previously, 2 μl of the forward primer and 2 μl of the reverse primer. The samples were placed in the thermocycler and incubated at 94°C for 5 min, followed by 35 cycles consisting of 94°C for 45 s, 57°C for 45 s, 72°C for 45 s, followed by 5 min of 72°C in order to denature, anneal and extend the sample, respectively. The PCR products were run on 1% agarose gel electrophoresis with 0.01% ethidium bromide. The gel was analysed using a gel imager (FluorChemE, Cell Biosciences, San Jose, CA). The bands of the PCR products were cut out under UV light exposure, and the cDNA products were isolated using a commercially available kit (PureLink™ Quick Gel Extraction Kit, ThermoFisher Scientific) following manufacturer instruction. The purified cDNAs were submitted for Sanger sequence analysis (GENEWIZ; South Plainfield, NJ, USA) to compare canine genetic sequences at the GenBank.

**TABLE 1:** Primer sequence and predicted product size

Target gene	Forward primer	Reverse primer	Product size (bp)
GAPDH	TCCATCTTCCAGGAGCGAGA	ATACATTGGGGGTGGGGACA	499
IBA‐1	ACCTGTCTACCGGCCTCTCC	AGCCCCATAAATCCCACCAC	519
CD204	CGGACCCCCAGGTGAAAAAG	CCAGCATCTTCCGCATGTGA	522

### 2.6 Western blot

Western blot was performed on the DH82 cell line to validate the protein expression of CD204 and IBA‐1. The DH82 cells were treated with 1 ml of cell lysis and extraction buffer (RIPA Buffer, ThermoFisher, Rockford, IL) and then incubated for 30 min at 4°C. The cells were then scraped and placed into a 1 ml microcentrifuge tube, homogenised with a 25‐gauge needle and incubated at 4°C for an additional 30 min. The sample was centrifuged at 8000 rpm for 10 min at 4°C and the supernatant was collected. The proteins were quantified using BCA concentration assay (Pierce Biotechnology, Rockford, IL) according to manufacturer's instructions. Thirty micrograms of protein were loaded onto 10% acrylamide gel (TGX Stain Free FastCast Acrylamide Starter Kit, 10%, Bio‐Rad, Hercules, CA, USA), prepared as per the manufacturer's instructions and then transferred to polyvinylidene difluoride (PVDF) membranes. The membranes were blocked with 2.5% ECL blocking agent (GE Healthcare, Buckinghamshire, UK) at 4°C overnight. Samples were probed with either IBA‐1 (1:1000, monoclonal mouse antibody, MiliporeSigma, Burlington, MA, USA) or CD204 (1:1000, monoclonal mouse antibody, Transgenic Inc, Kobe, Japan) antibodies, or with GAPDH (1:3000, monoclonal mouse antibody, Abcam, Cambridge, MA, USA), which served as a loading control. Each primary antibody was incubated 1 h in room temperature. The corresponding horseradish peroxidase conjugated secondary antibody (1:3000, goat anti‐mouse IgG‐HRP, Santa Cruz Biotechnology) was utilised in detection of IBA‐1, CD204 or GAPDH expression and was incubated for 1 h at room temperature. The proteins were then visualised using a commercial detection kit (ECL Prime Western Blotting Detection Reagent, GE Healthcare). Detection of signal was imaged with a gel imager (FluorChem E, Cell Biosciences, San Jose, CA, USA).

### 2.7 Immunohistochemistry

IHC was performed on the DH82 cell pellets and on the FFPE clinical tissues using the automated Leica BOND RX platform. Briefly, the slides were heated at 60°C for 10 min, then deparaffinised on the BOND RX using Dewax (Leica Biosystems, Buffalo Grove, IL, USA). The solution is heated to 72°C for 30 s. The slides are then rinsed with three changes of 100% reagent alcohol. They were rinsed in Bond Wash for 3 min, and then rinsed with DI water for 4 min. The slides were then treated with ER1 (antigen retrieval buffer) at 100°C for 20 min. The samples were treated with hydrogen peroxide and incubated for 10 min, and then rinsed with Bond Wash three times, for 2 min each. The slides were treated with identical mouse monoclonal primary antibody for either IBA‐1 (1:300, MiliporeSigma) or CD204 (1:200, Transgenic Inc), which was used in western blot above, and incubated for 15 min (Kato et al., 2013; Pierezan et al., 2014). The slides were then rinsed three times in Bond Wash, for 2 min each, then treated with Post Primary (Bond Polymer Refine Detection Kit, Leica Biosystems, Buffalo Grove, IL, USA), and then incubated for 15 min. The slides were then developed using DAB (3,3′‐diaminobenzidine tetrahydrochloride hydrate) (Bond Polymer Refine Detection Kit, Leica Biosystems, Buffalo Grove, IL, USA) and allowed to incubate for 10 min. The slides were then rinsed for 5 min in deionised water, counterstained with haematoxylin and then rinsed in tap water for 5 min. Lastly, the slides were mounted with a mounting medium (ClearVue Mountant XYL, Thermo Fisher, Waltham, MA, USA). To allocate an expression score, five randomly selected positive stained areas were counted. Semi‐quantitative evaluation scores ranged from 0 to 3: negative (score 0) = no positive cells; weakly positive (score 1) = 10–20% positive cells; moderately positive (score 2) ≤ 50% positive cells; strongly positive (score 3) ≥ 50% positive cells (Ide et al., 2011; Pierezan et al., 2014; Thongtharb et al., 2016). The negative control slides were treated with isotype control antibody (mouse IgG3, K: BD Bioscience, San Jose, CA, USA) with corresponding secondary antibody. All slides were reviewed by a single board‐certified anatomic pathologist (M,D).

### 2.8 RNA in situ hybridisation analysis

RNA ISH was performed on the DH82 cell line as well as archived FFPE tissue samples, 8 HS and 10 tumour types commonly included in the DDx of HS. RNAScope® target probes were designed by Advanced Cell Diagnostics (Advanced Cell Diagnostics, Newark, CA, USA). Chromogenic ISH was performed on a Leica BOND RX Fully Automated Research Stainer (Leica Biosystems, Buffalo Grove, IL, USA) using RNAscope technology. Automated, single‐plex, chromogenic RNAscope was performed using IBA‐1 (Cat No. 533898, CI‐AIF1) and CD204 (Cat No. 573901, CI‐MSR1) ISH probes. Pre‐treatment, hybridisation, signal amplification and detection were performed on the Leica BOND RX. Positive control slides were prepared with POLR2A (Cat No. 310988, CI‐POLR2A) and negative control slides were prepared with DapB (Cat No. 312038, Negative Control Probe_DapB), respectively (supplemental data. S1A‐D). Pre‐treatment conditions included sequential deparaffinisation, target retrieval using Leica Epitope Retrieval Buffer at 95°C for 20 min, protease digestion at 40°C for 15 min and endogenous enzyme block. Probes were hybridised at 42°C for 120 min, followed by signal development. After hybridisation, serial signal amplification reactions were followed by fast red chromogenic visualisation. Slides were counterstained with haematoxylin and ImmunoHisto‐Sealer (Diagnostic BioSystems, Pleasonton, CA, USA) was applied. Glass slides were visualised on an Olympus BX43 microscope (Olympus Corporation, Tokyo, Japan); photomicrographs were captured with a Spot Insight 12 Mp sCMOS Color Camera (Spot Imaging, Sterling Heights, MI, USA) and SPOT Imaging software (v5.4.3; Spot Imaging, Sterling Heights). The mRNA expression was scored using a standard ACD scoring system (Table 2) (A Guide for RNAscope Data Analysis: Advanced Cell Diagnosis Manual, 2021). Five randomly selected areas were counted for assessment with 40× magnification, following manufacturer's recommendation. High cellularity areas were considered as proper evaluation sites, and if significant cellular effects such as necrosis or haemorrhage were seen, they were avoided for the evaluation. In this study, we defined all HS samples as positive in our methods if they were scored as a 1, 2 or 3.

**TABLE 2:** RNAscope^®^ ISH scoring system of semi‐quantitative target gene expression analysis (ACD) (Wolff et al., 2018)

ACD Scoring	Numbers of dots/cell
Score 0	No staining or less than 1 dot in every ten cells
Score 1	1–3 dots/cell
Score 2	4–10 dots/cell
Score 3	10‐15 dots/cell and/or < 10% dots are in clusters
Score 4	>15 dots/cells and/or > 10% dots are in clusters

*Note*: Relative target gene expression is evaluated by counting numbers of clear dots per cell.

## 3 STATISTICS

In order to evaluate the mRNA expression of IBA‐1 and CD204 on 8 HS and 10 tumour types commonly included in the DDx of HS FFPE archived samples, we counted numbers of clear dots per cell on each sample. The mRNA expression scores between IBA‐1 and CD204 on 8 HS archived samples were compared using the Mann–Whitney *U* test. *p* value < 0.05 was considered as significant. Statistical analysis was performed using the GraphPad Prism 8 (GraphPad Software Inc., La Jolla, CA, USA).

## 4 RESULTS

### 4.1 mRNA expression of IBA‐1 and CD204 in DH82 cell line

**FIGURE 1:**
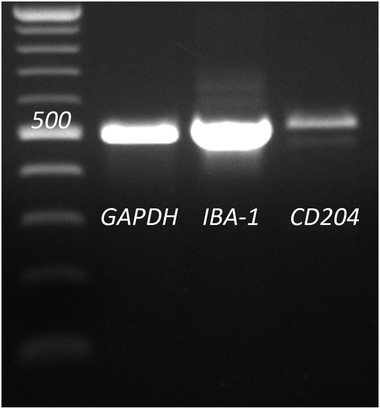
Results of RT‐PCR of GAPDH (housekeeping gene), IBA‐1 and CD204 in the DH82 cell line. Clear bands were obtained for all three targets; however, the target band of CD204 was of a diminished intensity compared to the other bands of GAPDH and IBA‐1. The size of each band matches the size predicted by the primers

RT‐PCR was performed to confirm the mRNA expression of IBA‐1 and CD204 in the DH82 cell line. Bands were detected for both target genes (Figure 1). GAPDH, the reference gene, was also detected in the DH82 cell line. The PCR products were sequenced, and canine specific IBA‐1 and CD204 expression was confirmed.

### 4.2 Protein expression in DH82 cell line

**FIGURE 2:**
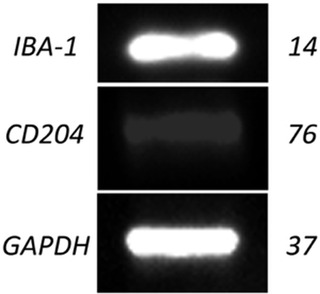
The protein expression of GAPDH, IBA‐1 and CD204 in the DH82 histiocytic cell line. GAPDH and IBA‐1 were detected at a similar high intensity, while CD204 expression was lower

Western blot was performed to confirm protein expression of IBA‐1 and CD204 in the DH82 cell line. Bands were detected at the expected values for all targets: 14 kDa for IBA‐1, 76 kDa for CD204 and 37 kDa for GAPDH, the loading control (Figure 2), confirming protein expression of IBA‐1 and CD204 in the DH82 cell line.

### 4.3 Protein expression of IBA‐1 and CD204 in cell pellets and archived clinical FFPE samples

IHC for IBA‐1 and CD204 was performed in the DH82 cell line pellets to validate both antibodies. Strong positive cytoplasmic/membrane expression of the markers was observed in these samples (Supplemental Data S2A‐D).

**FIGURE 3:**
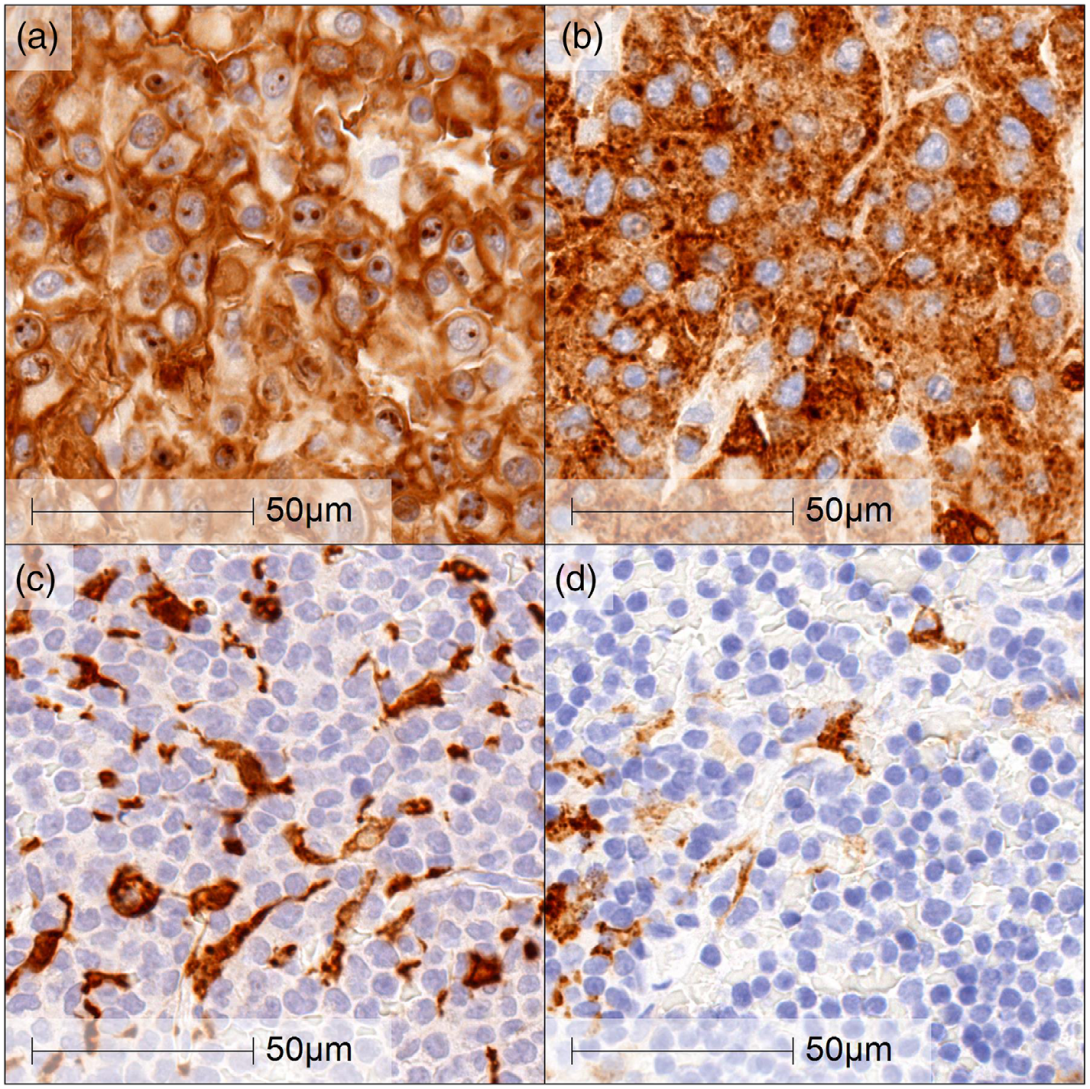
Immunohistochemistry on HS and mast cell tumour (common differential diagnosis: DDx). Cytoplasmic and membrane staining were seen using with IBA‐1 (a and c) and CD204 (b and d) antibodies. High intensity and more than 70% staining area were seen on this HS sample (a and b, score 3). On the other hand, scattered staining was seen on this mast cell tumour, which suggested macrophage infiltration (c and d). Scale bar indicates 50 μm

IHC was performed on archived 8 HS and 10 other selected tumour types, as described above, to verify and visualise the expression of IBA‐1 and CD204. The targets were visualised using a brown chromogen. The majority of the HS samples showed both positive cytoplasmic and membrane staining pattern and high intensity staining with IBA‐1 and CD204 (Figure 3a and b). IBA‐1 showed a score 3 on all 8 HS samples. CD204 showed a score 3 on 6 HS samples and a score 2 on 1 HS sample, but 1 sample did not show positivity on CD204 (Table 3). In the samples of lymphomas, mast cell tumours, melanomas, plasma cell tumours and anaplastic sarcomas, immunoreactivity for IBA‐1 or CD204 was absent in the neoplastic cells. Scattered positive non‐neoplastic macrophages were noted infiltrating the tumour stroma (Figure 3c and d).

**TABLE 3:** IHC and RNA‐ISH positivity result on 8 histiocytic sarcoma and 10 other types of tumours including common differential diagnosis (DDx)

	IHC		RNA‐ISH	
Type of tumour	IBA‐1	CD204	IBA‐1	CD204
HS	8/8	7/8	8/8	8/8
Other types of tumours	0/10	0/10	0/10	0/10

*Note*: One HS sample did not show positivity on CD204.

### 4.4 In situ expression of mRNA for IBA‐1 and CD204 genes

IBA‐1 and CD204 mRNA expression was assessed in the DH82 cell line pellets via the RNA‐ISH method to validate both RNAscope® probes (Supplemental Data S3A and B).

**FIGURE 4:**
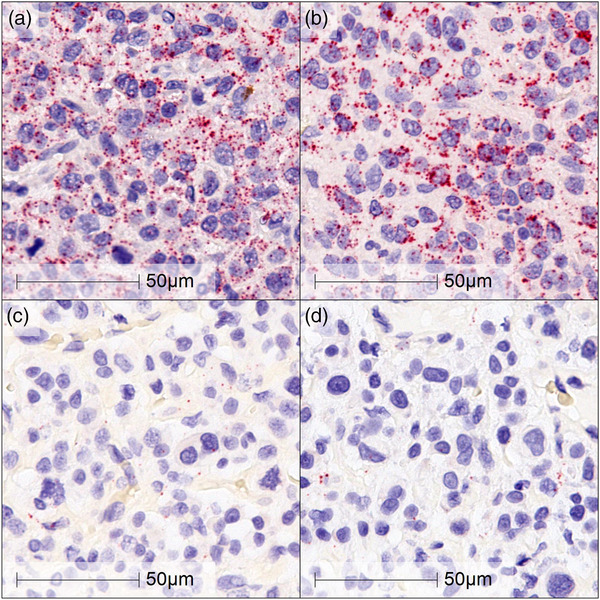
The mRNA expression of IBA‐1 and CD204 in archived HS (a and b) and plasma cell tumour (c and d). The samples probed with IBA‐1 (a and c) and CD204 (b and d) displayed high mRNA expression on HS. RNA‐ISH of IBA‐1 and CD204 showed 10–15 dots/cell and/or <10% dots are in clusters (ACD score: 3). On the other hand, there is minimum scattered staining on plasma cell tumour (common differential diagnosis: DDx). Scale bar indicates 50 μm

**FIGURE 5:**
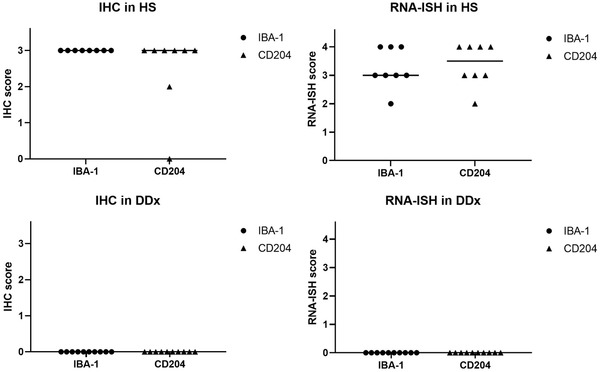
IHC and RNA‐ISH Scoring outcomes on 8 HS and 10 other types of tumours including common differential diagnosis (DDx) of HS archived samples. No significance staining score deference was seen IBA‐1 and CD204 mRNA expression with RNA‐ISH on 8 HS (IBA‐1: median was 3, CD204: median was 3.5). High IHC score and high staining RNA‐ISH score were seen on 8 HS with both IBA‐1 and CD204. Low IHC score and low staining RNA‐ISH score were seen on 10 other types of tumours including common DDx with both IBA‐1 and CD204

RNA‐ISH was also performed on 8 HS and 10 other selected tumour types, as described above. IBA‐1 and CD204 mRNA dots were detected in all 8 HS samples (Figure 4a and b). They were clearly visualised as punctate red dots, suggesting that IBA‐1 and CD204 are strong markers for canine HS. On the other hand, little and scattered staining was seen in all other tumour types, suspected to be macrophage infiltration (Figure 4c and d). The IBA‐1 and CD204 mRNA expression was evaluated using the ACD RNAscope® ISH scoring system as a semi‐quantitative gene expression analysis. Relative target gene expression was evaluated by manually counting the number of clear dots per cell. Most FFPE HS clinical samples showed a high level of IBA‐1 mRNA expression (score 4 *n* = 3, score 3 *n* = 4 and score 2 *n* = 1). Similarly, CD204 mRNA was found to have a high level of expression in the HS samples (score 4 *n* = 4, score 3 *n* = 3 and score 2 *n* = 1) (Figure 5, Table 3). No significant scoring difference was seen between IBA‐1 and CD204 expression in HS.

## 5 DISCUSSION

The present study reports the validation of an in situ analysis of IBA‐1 and CD204 mRNA expression in retrospective FFPE samples of canine histiocytic sarcoma. In this project, we elected to apply a semi‐quantitative RNA‐ISH method to assess expression of these markers in the neoplastic cells. We found that expression of the mRNAs for IBA‐1 and CD204 was high in most HS samples. In addition, a strong correlation was observed between the IHC score and the RNA‐ISH results. None of the neoplastic cells in our control tumours showed immunoreactivity for CD204 or IBA‐1, confirming that IBA‐1 and CD204 are additional useful markers in the diagnosis of canine HS. Scattered non‐neoplastic cells were observed to show IBA‐1 or CD204 protein or mRNA expression in the non‐HS tumour samples, likely consistent with a macrophagic infiltration. These markers’ specificity for cells of the monocyte/macrophage lineage is in correlation with previous published reports (Ide et al., 2011; Pierezan et al., 2014; Thongtharb et al., 2016). One histiocytic FFPE HS sample did not show positive immunoreactivity for CD204 during our IHC analysis but was positive on IHC for IBA‐1. Positive and negative IHC controls showed adequate results. The same FFPE HS sample did show positive labelling for CD204 mRNA upon RNA‐ISH. We suspect that the negative IHC labelling observed could be related to the IHC protocol and processing (Ramos‐Vara, 2005). It is also possible that, in this HS tumour sample, the CD204 protein may not be detectable with the IHC antibodies used due to post translational modifications or aberrant protein processing (Liu et al., 2016; Qin et al., 2020; Van Drie, 2011).

RNAscope® is compatible with routine formalin‐fixed, paraffin‐embedded tissue specimens and can allow for multiplex analysis. In addition, RNAscope® analysis for IBA‐1 or CD204 can be applied in cytological samples, which may be useful in the clinical setting as it may provide a definitive diagnosis of HS using a small sample such as a fine needle aspirate (Shiomitsu et al., 2020).

The RNA ISH method brings the benefits of in situ analysis and quantification of RNA biomarkers and can be supplemental to existing IHC methods if no antibodies are available (Wang et al., 2012). In our study, we elected to assess mRNA expression applying a manual count of the dots visualised through RNA ISH. We chose to use the suggested semi‐quantitative scoring system in which the number of dots per cells is recorded and associated with a specific score, as long as the positive control is expressed in adequate level (A Guide for RNAscope Data Analysis: Advanced Cell Diagnosis Manual, 2021). Our HS samples showed high scores of 2 and 3 using this method. Quantification of IBA‐1 and or CD204 mRNA expression via RNA‐ISH might be useful for the prediction of treatment response or prognosis, and we intend to assess expression scores with outcomes as we expand our selection of FFPE HS samples. A relative quantification could be applied utilising a dual detection RNA‐ISH method to compare the target gene expression with reference genes (Wolff et al., 2018). Manual enumeration of dots can be cumbersome; in the future, we intend to employ an automated measurement protocol using software analysis such as HALO software (Indica Labs) in order to improve the analytic performance and reduce human subjectivity in recognising the dots.

Due to the nature of our study (validation study), only a small number of FFPE tumour types were selected to act as possible negative samples. We selected cases of canine lymphomas, mast cell tumours, melanoma, plasma cell tumours and anaplastic sarcomas as these neoplastic processes can sometimes be misdiagnosed as HS. Recruiting additional tumour samples would help to confirm the specificity of the probes used. Another limitation would be unknown inter‐observer variability because of a single pathologist evaluation. It would be beneficial to involve more than 1 pathologist to minimise a potential inconsistency of the histopathological and immunohistological assessment.

## 6 CONCLUSION

While IHC remains a gold standard and widespread technique used in the diagnosis of canine HS, RNA‐ISH technology could be an additional useful diagnostic tool for identifying interested genes. This is the first study utilising RNA in situ hybridisation to identify distinguishing targets, IBA‐1 and CD204, to allow for the diagnosis of canine HS. We were able to validate the use of RNA‐ISH targeting IBA‐1 and CD204 in FFPE HS samples selected retrospectively and in our HS DH82 cell pellets. This molecular diagnostic technique can be paired with IHC to allow analysis of mRNA and protein, respectively, while also preserving the cellular relationship and tissue architecture.

## CONFLICT OF INTEREST

The authors have no conflict of interest.

## AUTHOR CONTRIBUTIONS

Keijiro Shiomitsu: conceptualisation; data curation; formal analysis; funding acquisition; investigation; methodology; project administration; resources; software; supervision; validation; visualisation; writing – original draft; writing – review & editing.

## Supporting information

SUPPORTING INFORMATION

SUPPORTING INFORMATION

SUPPORTING INFORMATION

## Data Availability

The data that support the findings of this study are available from the corresponding author upon reasonable request.

## References

[vms3795-bib-0001] https://www.Mbio.Ncsu.Edu/BioEdit/Bioedit.Html

[vms3795-bib-0002] A Guide for RNAscope Data Analysis: Advanced Cell Diagnosis Manual (2021). Retrieved from https://www.indicalab.com/wp‐content/uploads/2018/04/MK_51_103_RNAScope_data_analysis_guide_RevB.pdf. https://www.indicalab.com/wp‐content/uploads/2018/04/MK_51_103_RNAScope_data_analysis_guide_RevB.pdf

[vms3795-bib-0003] Affolter, V. K. , & Moore, P. F. (2002). Localized and disseminated histiocytic sarcoma of dendritic cell origin in dogs. Veterinary Pathology, 39(1), 74–83. 10.1354/vp.39-1-74 12102221

[vms3795-bib-0004] Asada, H. , Tomiyasu, H. , Goto‐Koshino, Y. , Fujino, Y. , Ohno, K. , & Tsujimoto, H. (2015). Evaluation of the drug sensitivity and expression of 16 drug resistance‐related genes in canine histiocytic sarcoma cell lines. Journal of Veterinary Medical Science, 77(6), 677–684. 10.1292/jvms.14-0415 25715778 PMC4488404

[vms3795-bib-0005] Barnes, A. , Bee, A. , Bell, S. , Gilmore, W. , Mee, A. , Morris, R. , & Carter, S. D. (2000). Immunological and inflammatory characterisation of three canine cell lines: K1, K6 and DH82. Veterinary Immunology and Immunopathology, 75(1), 9–25. 10.1016/S0165-2427(00)00184-7 10889296

[vms3795-bib-0006] Bingham, V. , McIlreavey, L. , Greene, C. , O'Doherty, E. , Clarke, R. , Craig, S. , Salto‐Tellez, M. , McQuaid, S. , Lewis, C. , & James, J. (2017). RNAscope *in situ* hybridization confirms mRNA integrity in formalin‐fixed, paraffin‐embedded cancer tissue samples. Oncotarget, 8(55), 93392–93403. Published online 2017. 10.18632/oncotarget.21851 29212158 PMC5706804

[vms3795-bib-0007] Chan, S. , Filézac de L'Etang, A. , Rangell, L. , Caplazi, P. , Lowe, J. B. , & Romeo, V. (2018). A method for manual and automated multiplex RNAscope in situ hybridization and immunocytochemistry on cytospin samples. PLoS One, 13(11), e0207619. 10.1371/journal.pone.0207619 30458053 PMC6245747

[vms3795-bib-0008] Erich, S. A. , Constantino‐Casas, F. , Dobson, J. M. , & Teske, E. (2018). Morphological distinction of histiocytic sarcoma from other tumor types in bernese mountain dogs and flatcoated retrievers. In Vivo (Athens, Greece), 32(1), 7–17. 10.21873/invivo.11198 PMC5892626 29275293

[vms3795-bib-0009] Fulmer, A. K. , & Mauldin, G. E. (2007). Canine histiocytic neoplasia: An overview. The Canadian Veterinary Journal, 48(10), 1041–1043, 1046–1050. 17987966 PMC1978291

[vms3795-bib-0010] Heinrich, F. , Contioso, V. B. , Stein, V. M. , Carlson, R. , Tipold, A. , Ulrich, R. , Puff, C. , Baumgärtner, W. , & Spitzbarth, I. (2015). Passage‐dependent morphological and phenotypical changes of a canine histiocytic sarcoma cell line (DH82 cells). Veterinary Immunology and Immunopathology, 163(1–2), 86–92. 10.1016/j.vetimm.2014.11.006 25534080

[vms3795-bib-0011] Ide, T. , Uchida, K. , Kagawa, Y. , Suzuki, K. , & Nakayama, H. (2011). Pathological and immunohistochemical features of subdural histiocytic sarcomas in 15 dogs. Journal of Veterinary Diagnostic Investigation, 23(1), 127–132. 10.1177/104063871102300123 21217043

[vms3795-bib-0012] Kato, Y. , Funato, R. , Hirata, A. , Murakami, M. , Mori, T. , Maruo, K. , Yanai, T. , & Sakai, H. (2014). Immunocytochemical detection of the class A macrophage scavenger receptor CD204 using air‐dried cytologic smears of canine histiocytic sarcoma. Veterinary Clinical Pathology, 43(4), 589–593. 10.1111/vcp.12190 25168797

[vms3795-bib-0013] Kato, Y. , Murakami, M. , Hoshino, Y. , Mori, T. , Maruo, K. , Hirata, A. , Nakagawa, T. L. D. R. , Yanai, T. , & Sakai, H. (2013). The class A macrophage scavenger receptor CD204 is a useful immunohistochemical marker of canine histiocytic sarcoma. Journal of Comparative Pathology, 148(2), 188–196. 10.1016/j.jcpa.2012.06.009 22901707

[vms3795-bib-0014] Liu, Y. , Beyer, A. , & Aebersold, R. (2016). On the dependency of cellular protein levels on mRNA abundance. Cell, 165(3), 535–550. 10.1016/j.cell.2016.03.014 27104977

[vms3795-bib-0015] Moore, P. F. , Affolter, V. K. , & Vernau, W. (2006). Canine hemophagocytic histiocytic sarcoma: A proliferative disorder of CD11d+ macrophages. Veterinary Pathology, 43(5), 632–645. 10.1354/vp.43-5-632 16966440

[vms3795-bib-0016] Pierezan, F. , Mansell, J. , Ambrus, A. , & Hoffmann, A. R. (2014). Immunohistochemical expression of ionized calcium binding adapter molecule 1 in cutaneous histiocytic proliferative, neoplastic and inflammatory disorders of dogs and cats. Journal of Comparative Pathology, 151(4), 347–351. 10.1016/j.jcpa.2014.07.003 25172051

[vms3795-bib-0017] Qin, H. , Ni, H. , Liu, Y. , Yuan, Y. , Xi, T. , Li, X. , & Zheng, L. (2020). RNA‐binding proteins in tumor progression. Journal of Hematology & Oncology, 13(1), 90. 10.1186/s13045-020-00927-w 32653017 PMC7353687

[vms3795-bib-0018] Ramos‐Vara, J. A. (2005). Technical aspects of immunohistochemistry. Veterinary Pathology, 42(4), 405–426. 10.1354/vp.42-4-405 16006601

[vms3795-bib-0019] Shiomitsu, K. , Bechtel, S. M. , Thompson, P. M. , & Frasca, S. (2020). Molecular diagnosis using RNAscope in‐situ hybridization in canine malignancies. Canadian Journal of Veterinary Research, 84(4), 319–323. 33012982 PMC7491000

[vms3795-bib-0020] Thongtharb, A. , Uchida, K. , Chambers, J. K. , Kagawa, Y. , & Nakayama, H. (2016). Histological and immunohistochemical studies on primary intracranial canine histiocytic sarcomas. Journal of Veterinary Medical Science, 78(4), 593–599. 10.1292/jvms.15-0627 26668164 PMC4873849

[vms3795-bib-0021] Van Drie, J. H. (2011). Protein folding, protein homeostasis, and cancer. Chinese Journal of Cancer, 30(2), 124–137. 10.5732/cjc.010.10162 21272445 PMC4013342

[vms3795-bib-0022] Wakahashi, K. , Shimoyama, M. , Katayama, Y. , Minagawa, K. , Yoshida, K. , Sasaki, R. , Nakayama, S. , Yokozaki, H. , Yanagita, E. , Itoh, T. , Hayashi, Y. , & Matsui, T. (2010). Histiocytic sarcoma with two immunohistopathologically distinct populations. International Journal of Hematology, 92(4), 642–646. 10.1007/s12185-010-0699-1 20924729

[vms3795-bib-0023] Wang, F. , Flanagan, J. , Su, N. , Wang, L.‐C. , Bui, S. , Nielson, A. , Wu, X. , Vo, H.‐T. , Ma, X.‐J. , & Luo, Y. (2012). RNAscope: A novel in situ RNA analysis platform for formalin‐fixed, paraffin‐embedded tissues. The Journal of Molecular Diagnostics, 14(1), 22–29. 10.1016/j.jmoldx.2011.08.002 22166544 PMC3338343

[vms3795-bib-0024] Wellman, M. L. , Krakowka, S. , Jacobs, R. M. , & Kociba, G. J. (1988). A macrophage‐monocyte cell line from a dog with malignant histiocytosis. In Vitro Cellular & Developmental Biology, 24(3), 223–229. 10.1007/BF02623551 3350786

[vms3795-bib-0025] Wolff, A. C. , Hammond, M. E. H. , Allison, K. H. , Harvey, B. E. , Mangu, P. B. , Bartlett, J. M. S. , Bilous, M. , Ellis, I. O. , Fitzgibbons, P. , Hanna, W. , Jenkins, R. B. , Press, M. F. , Spears, P. A. , Vance, G. H. , Viale, G. , McShane, L. M. , & Dowsett, M. (2018). Human epidermal growth factor receptor 2 testing in breast cancer: American Society of Clinical Oncology/College of American Pathologists Clinical Practice Guideline Focused Update. Journal of Clinical Oncology, 36(20), 2105–2122. 10.1200/JCO.2018.77.8738 29846122

