# Supplementary material for: RNA in situ hybridisation as a molecular diagnostic technique targeting IBA‐1 and CD204 in canine histiocytic sarcoma
Source: Vet Med Sci. 2022 Mar 26;8(4):1400–8. doi: 10.1002/vms3.795 (PMC9297782; doi:10.1002/vms3.795)
Supplement: Supplementary file 1 — SUPPORTING INFORMATION [file VMS3-8-1400-s003.docx]

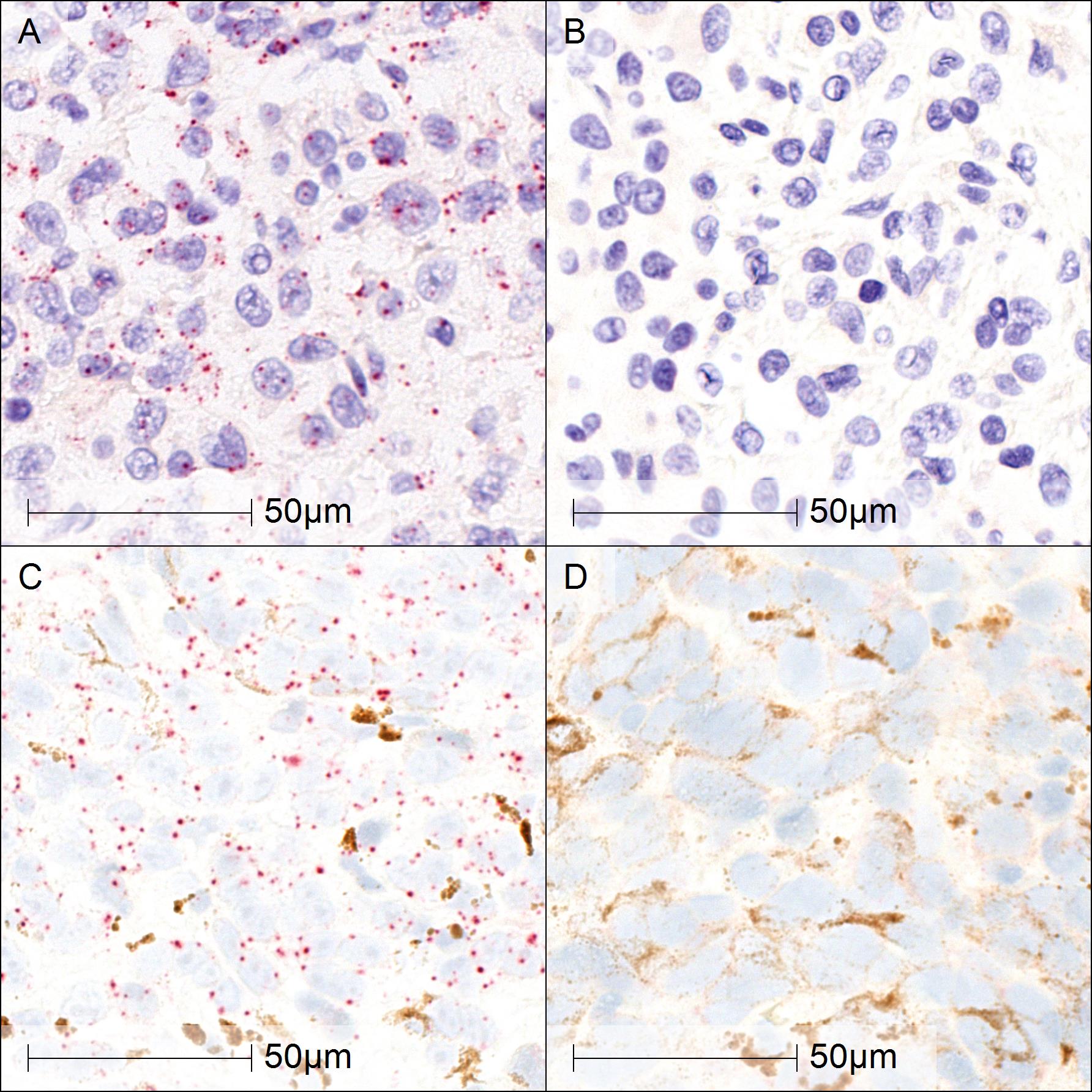


S1A-D: Positive (POLR2A: S1A and C) and negative (DapB: B and D) control RNA-ISH in HS (S1A and B) and melanoma (S1C and D). Targeted mRNA-probe hybridization reactions are visualized as clear red dots with some clusters. The samples probed with POLR2A showed high expression. The samples probed with DapB showed no expression. Scale bar indicates 50 µm.
