# Supplementary material for: RNA in situ hybridisation as a molecular diagnostic technique targeting IBA‐1 and CD204 in canine histiocytic sarcoma
Source: Vet Med Sci. 2022 Mar 26;8(4):1400–8. doi: 10.1002/vms3.795 (PMC9297782; doi:10.1002/vms3.795)
Supplement: Supplementary file 2 — SUPPORTING INFORMATION [file VMS3-8-1400-s001.docx]

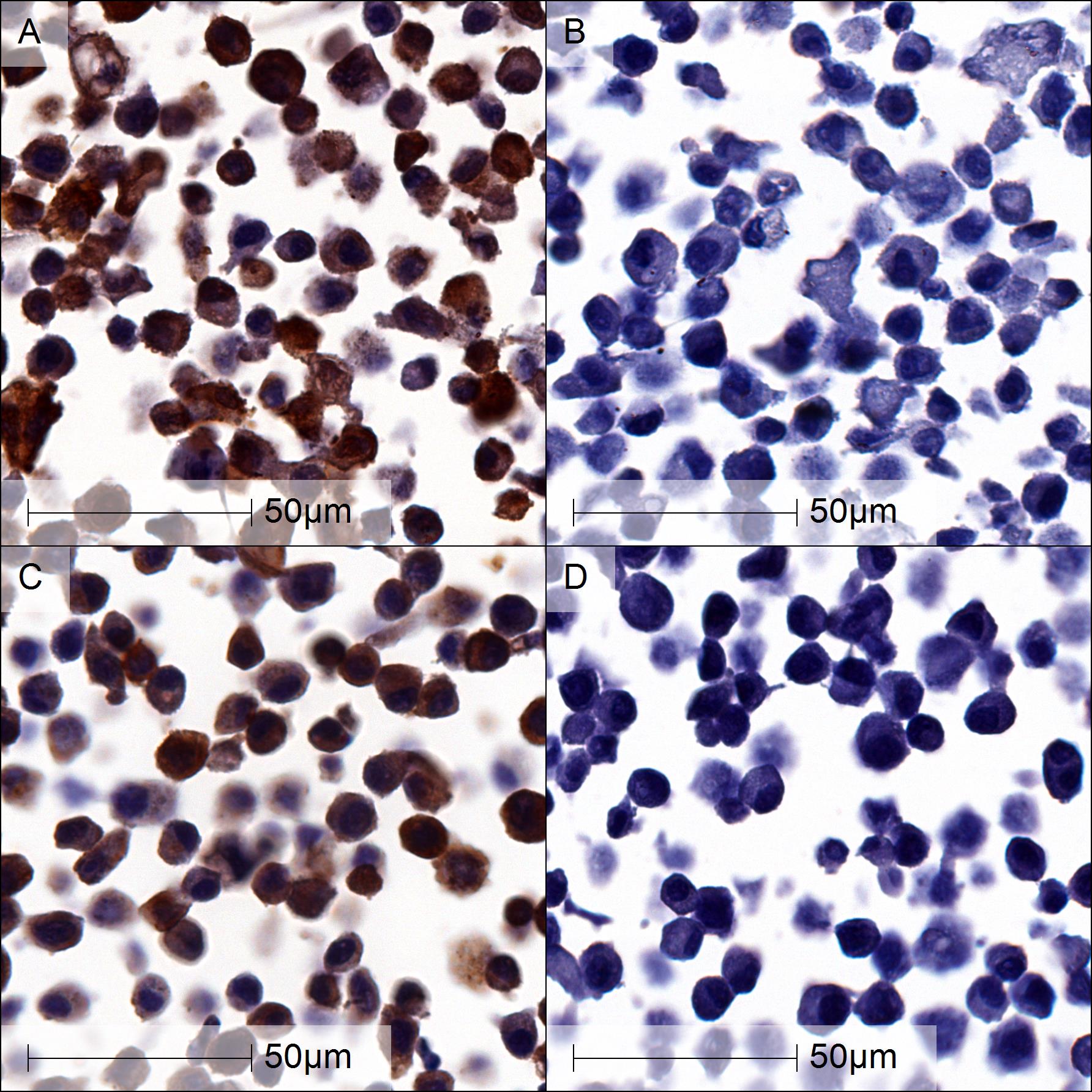


S2A-D: DH82 cell pellet; protein expression of IBA-1 when probed with anti-IBA-1 antibody (S2A) DH82 cell pellet; negative control (S2B) DH82 cell pellet; protein expression of CD204 when probed with anti-CD204 antibody (S2C) DH82 cell pellet; negative control (S2D). DH82 cell pellets showed high expression of IBA-1 and CD204. The negative control slides showed no reactivity. Scale bar is 50 μm.
