# Supplementary material for: RNA in situ hybridisation as a molecular diagnostic technique targeting IBA‐1 and CD204 in canine histiocytic sarcoma
Source: Vet Med Sci. 2022 Mar 26;8(4):1400–8. doi: 10.1002/vms3.795 (PMC9297782; doi:10.1002/vms3.795)
Supplement: Supplementary file 3 — SUPPORTING INFORMATION [file VMS3-8-1400-s002.docx]

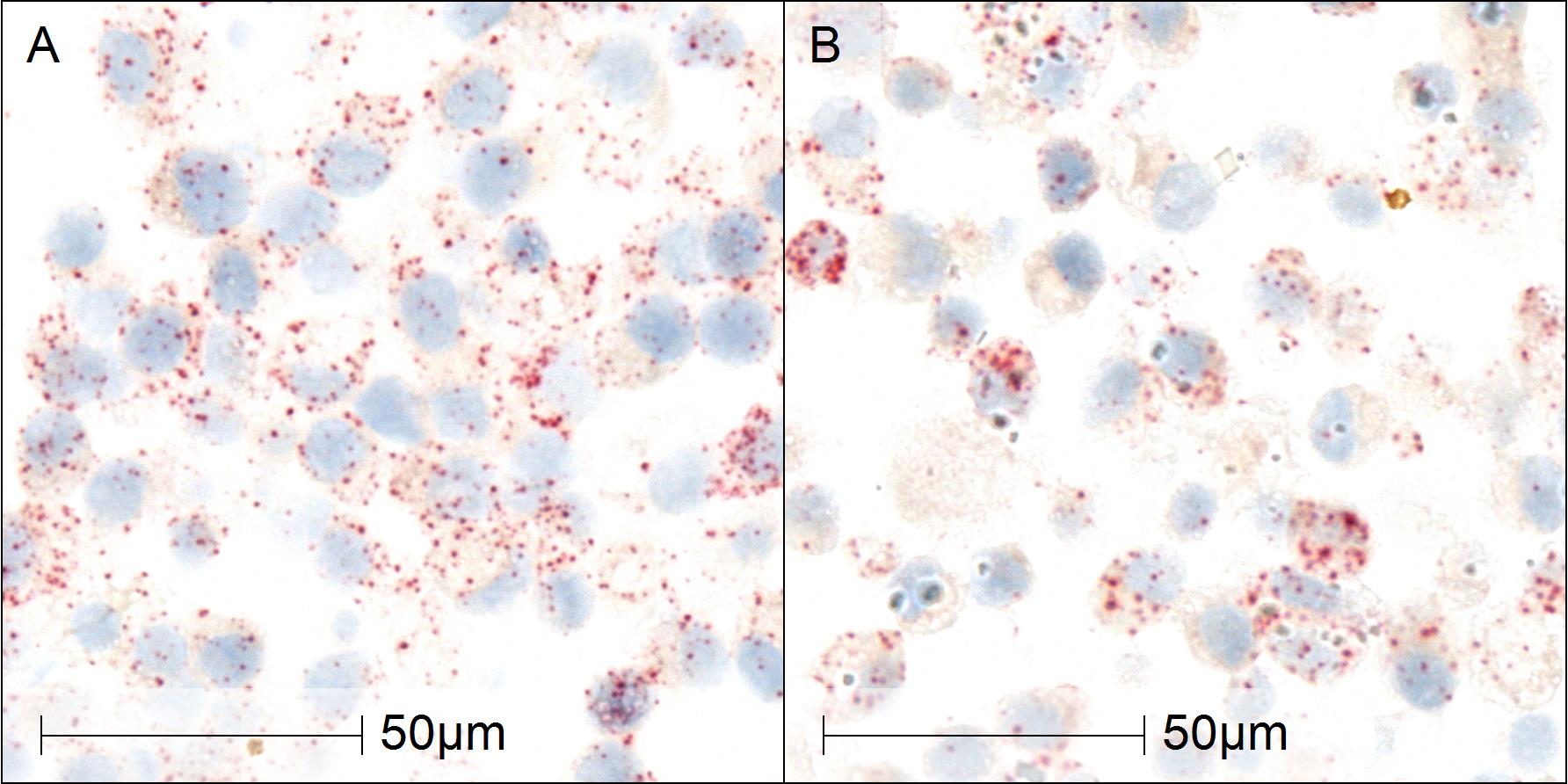


S3A and B: RNA-ISH: IBA-1 and CD204 on DH82 cell pellet. RNA ISH results using ZZ probes to mRNA of IBA-1 (S3A) and CD204 (S3B). Targeted mRNA-probe hybridization reactions are visualized as red dots clearly with some clusters. Scale bar is 50 μm.
